# Effects of Concurrent and Terminal Visual Feedback on Ankle Co-Contraction in Older Adults during Standing Balance

**DOI:** 10.3390/s21217305

**Published:** 2021-11-02

**Authors:** Rachel V. Vitali, Vincent J. Barone, Jamie Ferris, Leia A. Stirling, Kathleen H. Sienko

**Affiliations:** 1 Department of Mechanical Engineering, University of Iowa, Iowa City, IA 52242, USA; 2 Department of Mechanical Engineering, University of Michigan, Ann Arbor, MI 48109, USA; vbarone@umich.edu (V.J.B.); jcferris@umich.edu (J.F.); 3 Department of Industrial and Operations Engineering, University of Michigan, Ann Arbor, MI 48109, USA; leias@umich.edu; 4 Robotics Institute, University of Michigan, Ann Arbor, MI 48109, USA

**Keywords:** balance, visual feedback, sensory augmentation, older adult, wearable sensors, surface electromyography

## Abstract

This preliminary investigation studied the effects of concurrent and terminal visual feedback during a standing balance task on ankle co-contraction, which was accomplished via surface electromyography of an agonist–antagonist muscle pair (medial gastrocnemius and tibialis anterior muscles). Two complementary mathematical definitions of co-contraction indices captured changes in ankle muscle recruitment and modulation strategies. Nineteen healthy older adults received both feedback types in a randomized order. Following an analysis of co-contraction index reliability as a function of surface electromyography normalization technique, linear mixed-effects regression analyses revealed participants learned or utilized different ankle co-contraction recruitment (i.e., relative muscle pair activity magnitudes) and modulation (i.e., absolute muscle pair activity magnitudes) strategies depending on feedback type and following the cessation of feedback use. Ankle co-contraction modulation increased when concurrent feedback was used and significantly decreased when concurrent feedback was removed. Ankle co-contraction recruitment and modulation did not significantly change when terminal feedback was used or when it was removed. Neither ankle co-contraction recruitment nor modulation was significantly different when concurrent feedback was used compared to when terminal feedback was used. The changes in ankle co-contraction recruitment and modulation were significantly different when concurrent feedback was removed as compared to when terminal feedback was removed. Finally, this study found a significant interaction between feedback type, removal of feedback, and order of use of feedback type. These results have implications for the design of balance training technologies using visual feedback.

## 1. Introduction

It is well known that balance performance is negatively correlated with age, which increases the risk and, subsequently, the prevalence of falls in older adults. The literature reveals that as many as one in three adults 65 years and older fall at least once a year, and half of those adults fall multiple times [1]. Annually in the United States, approximately $50 billion is spent on medical costs related to non-fatal fall injuries [2]. Recent systematic reviews and meta-analyses have shown that balance training can improve balance performance among older adults [3,4]. Many balance training programs employ feedback systems (including sensors and displays) aimed at augmenting conventional balance training [4]. These sensory augmentation technologies have been studied as real-time balance aids as well as rehabilitation training aids to promote sensory reweighting [5]. Some of the sensing element technologies studied to date utilize expensive and specialized laboratory-based equipment for sensing postural sway (e.g., motion-tracking systems and force plates), which inevitably creates scalability roadblocks to widespread adoption, both in a clinical (out-patient) setting as well as in the home. For this reason, research groups have investigated alternative sensing approaches like wearable technologies [6,7].

The sensed information can be displayed using various modalities, including auditory [7], haptic [8], visual [9,10,11], or multimodal feedback [12,13]. It should be noted that each of these feedback modalities have been shown to yield decreased postural sway under certain nonperturbed and/or perturbed conditions. Visual feedback has been extensively implemented among healthy and older adults with various pathologies [9,13], as well as younger adults [14,15]. Visual feedback is straightforward for participants to integrate into their performance [4] and encodes spatial and temporal information that is critical to a stationary task like standing balance [16].

Visual feedback can be concurrent (i.e., real-time) or terminal (post-trial). The specificity of practice hypothesis posits that learning is specific to the source of information that is likely to ensure optimal performance [14]. Since older adults tend to favor visual information [17], concurrent visual feedback may lead to significant, short-term improvements [9], but these gains are rarely present during subsequent retention testing [4,9]. This trend can be explained by the guidance hypothesis, which suggests that feedback can have negative effects on performance if it is provided in a form that is too easy to use [18]. By removing feedback and/or transitioning to infrequent terminal feedback, these effects can be lessened since users are less dependent on the feedback [19,20]. However, differences in the effects of concurrent and terminal visual feedback (or their interaction [15]) on balance training performance are not well understood.

While most studies have focused on how visual feedback (concurrent or terminal) affects an outcome measure related to standing balance performance, it is not clear how these improvements were achieved. Standing balance performance is frequently quantified by center of mass or center of pressure deviations and/or velocities as measured by a force plate (e.g., [21]). Users incorporate this information into their standing balance strategy to decrease their deviations from upright [21]. The strategy that accomplished those reductions is not discernable from the available kinematic data. Electromyography (EMG) data (most frequently collected on the skin surface) can reveal how muscle recruitment and modulation change in parallel with improvements in standing balance performance.

Of interest to this work is the relationship between agonist–antagonist muscle (or muscle group) pairs. For example, when the tibialis anterior and gastrocnemius muscles contract simultaneously, they produce movements about the ankle that act in opposite directions from one another. The result can stiffen the joint based on the amplitude of the contraction, with the goal of reducing the amount of allowable moment [22,23]. Past research has shown this strategy of stiffening the ankle is not necessarily a successful one [5,24,25], especially for older adults who already require more muscle activity than younger adults to produce equivalent torques [26]. For example, Warnica et al. [25] found an increase in ankle co-contraction coincided with an increase in center of pressure deviation. While Kiemel et al. [27] showed that the central nervous system does not produce more muscle activation than is necessary to stabilize upright stance, young healthy participants have been shown to be able to reduce muscle activation without altering balance performance with auditory EMG feedback [7]. When older adults have been instructed to reduce their sway [24] or when they have perceived a postural threat [28], they have increased their ankle co-contraction without meaningful reductions in their sway. Fall risk in older adults has also been positively correlated with co-contraction [29]. Thus, increased ankle co-contraction is considered to be a nondiscriminatory (i.e., utilized regardless of balance condition) and largely detrimental strategy adopted by older adults to improve standing balance performance [30].

This study investigated how concurrent and terminal visual feedback affected ankle co-contraction in older adults during a standing balance task. This study also examined two common, but physiologically different, co-contraction definitions to better understand participants’ strategies. This study aimed to address the following hypotheses: (**H1**) ankle co-contraction would increase relative to baseline when either type of feedback is used; (**H2**) ankle co-contraction would decrease when concurrent feedback is removed relative to when the feedback was used; (**H3**) ankle co-contraction would decrease when terminal feedback is removed relative to when the feedback is used, but the decrease would be less than with concurrent feedback, ultimately leading co-contraction after training to be higher with concurrent feedback than terminal feedback; and (**H4**) increases in ankle co-contraction relative to baseline would be smaller for terminal feedback than for concurrent feedback.

## 2. Materials and Methods

### 2.1. Participants

A convenience sample of 19 older adults (65–80 years old; 7 males, 12 females) were recruited to participate in this preliminary study investigating the effects of two types of feedback. Inclusion criteria included general good health and no history of muscular or neurological disorders. The study was approved by the University of Michigan’s Institutional Review Board (HUM00015990). All participants gave written informed consent in accordance with the Declaration of Helsinki [31].

### 2.2. Experimental Protocol

The experiment was a crossover design, in which participants completed both the concurrent and terminal feedback conditions on the same day. They were randomly assigned to either the concurrent feedback testing block first or terminal feedback testing block first (see Table 1). All participants performed the same standing balance task with their feet together on a foam pad (balance pad, 50 × 41 × 6 cm, Airex AG, Sins, Switzerland). This task was sufficiently challenging enabling healthy older adults the opportunity to improve their balance performance with the aid of feedback.

**Figure 1:**
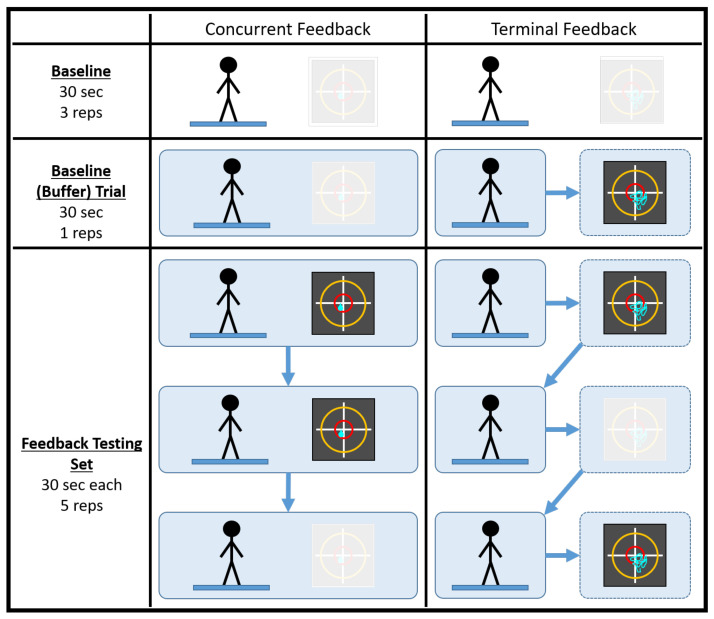
An illustration of how a generic testing session was conducted. The baseline (buffer) trial acted as the fourth and final baseline trial for the feedback block of testing. The black squares represent the visual feedback displays utilized in the study. The greyed out visual display denotes the cases when feedback was not used.

A testing session consisted of 38 trials, half of which were conducted in a concurrent feedback testing block and half of which were conducted in a terminal feedback testing block. It should be noted that the testing session duration was comparable to a typical single-day balance therapy or at-home balance training session. Prior to data collection, participants practiced with the type of feedback they would be receiving while adopting a different stance (feet shoulder width apart) on a different surface (firm) for, at most, 60 s. Each feedback testing block consisted of four baseline trials (no feedback) and five feedback testing sets of three 30 s trials. For each set, feedback was provided for the first two trials and was removed for the third. Figure 1 below illustrates how a generic testing session was conducted. Participants took a short break between the feedback testing blocks to minimize the effects of fatigue. It should also be noted that, prior to a deliberate change in experimental protocol, the first seven participants did not complete their final trial during the terminal feedback condition or the fourth baseline (buffer) trial during the concurrent feedback condition.

Visual feedback was displayed on a projector screen located 10 feet in front of the participant, such that the center of the display was approximately level with the participant’s line of sight. A pair of horizontal and vertical axes represented the medial–lateral (ML) and anterior–posterior (AP) sway directions, respectively, and sway angles were estimated by an inertial measurement unit (IMU; MTx, XSens Inc, Eschende, The Netherlands) on an elastic belt positioned just above the sacrum. Concurrent feedback was displayed as a single cursor on the screen denoting the current ML and AP sway angles of the participant. Terminal feedback was displayed at the end of a trial as a stabilogram illustrating the entire trial’s sway angle trajectory. A previous investigation compared the effects of feedback type and removal on outcome measures of balance performance derived from the IMU data [32].

### 2.3. Surface Electromyography

Surface electromyography (sEMG) was collected from both legs from two bilateral muscles—tibialis anterior and medial gastrocnemius. The medial gastrocnemius was chosen as the most appropriate muscle for sEMG as it is the most superficial plantar flexor that is relatively easy to palpate and place in an older population (as compared to the soleus, for example). The Delsys DS-B04 Bagnoli-16 EMG system with a SP-B08 Bagnoli-16 main amplifier was used in the study. Sensors were placed according to the Delsys Bagnoli system manual, with the contacts perpendicular to the muscle fibers. Electrode placement locations were cleaned with alcohol wipes and placements targeted the center of the muscle. The sEMG sensor contacts were made from 99.9% pure silver bars measuring 10 mm in length and 1 mm in diameter, and were spaced 10 mm apart for optimal signal detection and consistency. The single differential sEMG sensors were affixed with Bagnoli adhesive sensor interfaces made of medical adhesive. The system collected synchronized sEMG data at 1000 Hz. All sEMG data underwent bandpass filtering (second-order Butterworth with cutoff frequencies of 30 Hz and 400 Hz) to remove movement artifacts and high-frequency noise components. After full wave rectification, linear envelopes were extracted via low-pass filtering (fourth-order Butterworth with a cutoff frequency of 6 Hz).

It is standard practice, particularly when calculating co-contraction indices as described next, to normalize the sEMG signals. Since the experimental protocol did not include a maximum voluntary contraction (MVC) task, two other normalization techniques that are regularly utilized in the literature [33] were selected. The first technique was to normalize a specific muscle’s sEMG time series by the maximum or peak value demonstrated by that specific muscle across all trials of the same task on the same day. The second technique was to normalize a muscle’s sEMG time series by the average or mean activation level demonstrated across all trials of the same task on the same day. Evidence suggests that these options are either comparable in terms of reliability [34] or that the mean is slightly superior [35]. Both normalization techniques were utilized and evaluated, as further discussed in Section 2.5.

### 2.4. Co-Contraction Index

There does not exist a universally accepted mathematical definition for calculating a co-contraction index (CCI) for an agonist–antagonist muscle pair. Two of the most extensively used definitions for CCI were proposed by Falconer and Winter [36], and Rudolph, Axe, and Snyder-Mackler [37], though the latter definition is frequently attributed to Lewek, Rudolph, and Snyder-Mackler [38]. Both definitions have been slightly modified here for the sake of consistency. The Falconer and Winter (FW) CCI definition is

(1) $$CCI_{FW}=\frac{1}{n}\overset{n}{\underset{i=1}{\sum }}\frac{2\times sEMG_{low,i}}{sEMG_{low,i}+sEMG_{high,i}}\times 100\%$$

where $sEMG_{low,i}$ denotes the sEMG value for whichever muscle is smaller in magnitude at an instant in time, $sEMG_{high,i}$ denotes the sEMG value for whichever muscle is larger in magnitude at an instant in time, and *n* is the total number of samples. The ‘2’ in the numerator accounts for the additional force the agonist muscle must produce to counteract the effort expended by the antagonist muscle. $CCI_{FW}$ can range from 0%, denoting the case when the antagonist muscle is not contracting at all, to 100%, denoting the case when the antagonist muscle is contracting the equivalent amount as the agonist muscle. While this definition of CCI is well suited to quantifying the relative amount of antagonist muscle activity, it would report a high level of co-contraction regardless of how much the muscles are activated. For a relatively dynamic activity like gait, this has been shown to be problematic when using CCI as a proxy for joint stiffness [39,40].

On the other hand, the Rudolph/Lewek (RL) definition is

(2) $$CCI_{RL}=\frac{1}{n}\overset{n}{\underset{i=1}{\sum }}\left(\frac{sEMG_{low,i}}{sEMG_{high,i}}\right)\times \left(sEMG_{low,i}+sEMG_{high,i}\right)\times 100\%$$

where $sEMG_{low}$ denotes the sEMG value for whichever muscle is smaller in magnitude for an instant in time, $sEMG_{high}$ denotes the sEMG value for whichever muscle is larger in magnitude for an instant in time, and *n* is the total number of samples. $CCI_{RL}$ can range from 0%, again denoting the case when the antagonist muscle is not contracting, to 200%, denoting the case when the antagonist muscle is contracting the equivalent amount as the agonist muscle. Unlike $CCI_{FW}$, this definition of CCI is the product of two terms containing different kinds of information. The first term is a ratio between the agonist and antagonist muscle activation, whereas the second term is the sum of the total amount of muscle activation.

**Figure 2:**
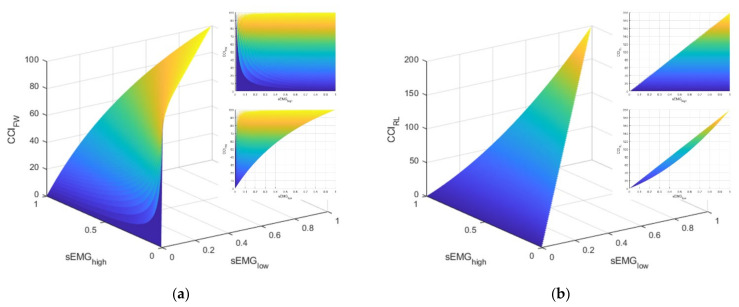
This figure illustrates the manifolds for the co-contraction indices (CCI) based on the definitions described by: (**a**) Falconer and Winter (FW) and (**b**) Rudolph/Lewek (RL). The callout in the upper right of each subfigure is a view of the manifold in the sEMG_high_–CCI plane. The callout directly below is a view of the manifold in the sEMG_low_–CCI plane.

As elucidated above, these two CCI definitions have notably different physiological interpretations. The FW definition is blind to the total level of activation exhibited by the muscles. However, with a somewhat homogenous task like standing balance, it is unclear whether these differences would be meaningful. Furthermore, the literature reveals both definitions have been used to evaluate ankle muscle strategies during standing balance tasks (e.g., [41] used the FW definition and [5] used the RL definition). Let us consider the interpretations and behaviors for each CCI definition. Figure 2 illustrates manifolds describing the possible values of CCI given different values for agonist ($sEMG_{high}$)–antagonist ($sEMG_{low}$) muscle pair values. Note that these manifolds were produced with simulated data and, by definition, $sEMG_{low}$ cannot be greater than $sEMG_{high}$.

Apart from the difference in the domains of the CCI values (i.e., [0, 100] for FW and [0, 200] for RL), consider the markedly different relationships between $sEMG_{high}$ and the CCI for the two definitions (i.e., the topmost callout in the upper right of each subplot in Figure 2). For the FW definition, the CCI can take on any value in its domain because the definition is driven by the value of $sEMG_{low}$. In other words, the FW definition of CCI is only providing information about the contribution of the antagonist muscle relative to the contribution of the agonist muscle. This characteristic is in contrast with the RL definition, which has an upper limit for the CCI that is regulated by how much muscle activation is present. Next, consider the strikingly different relationships between $sEMG_{low}$ and the CCI. For the RL definition, the domain for CCI is limited in the sense that larger values for CCI are primarily achieved by increasing the value of $sEMG_{low}$, which, by definition, means $sEMG_{high}$ must increase as well. By contrast, larger values of the FW definition of CCI can be achieved by lower values of $sEMG_{high}$ when $sEMG_{low}$ is small. Consequently, the lower limit of CCI is guided by the magnitude of $sEMG_{low}$. Both CCI definitions are utilized and evaluated, as discussed next.

### 2.5. Statistical Analyses

Past research has provided evidence that normalizing sEMG signals by the mean activity level for a task is either comparable or may be more reliable than normalizing by the peak activity level [33,34,35]. However, given the definitions of CCI documented in the previous section, it is possible that the approaches to calculating CCI could be sensitive to the normalization technique. For example, $CCI_{RL}$ explicitly considers the total amount of muscle activation, which will be different depending on the normalization technique. Prior to the statistical analyses evaluating the effects of feedback, interclass coefficients (ICCs) for each of the normalization techniques and CCI definitions were calculated to assess reliability. Specifically, ICC calculations and the corresponding 95% confidence intervals were computed using custom scripts in R with the psych package based on a mean-rating (k = 3), absolute-agreement, 2-way mixed effects model (i.e., ICC(3,k)). ICC scores were interpreted via widely used guidelines [42]. Specifically, ICC values that are less than 0.5 are poor, between 0.5 and 0.75 are moderate, between 0.75 and 0.9 are good, and above 0.9 are excellent. For this calculation, the first three baseline trials for the testing session for each participant were included to provide multiple measures for the same task. Each leg was analyzed separately and then their ICC values were averaged. 

To address the hypotheses outlined above, a linear mixed-effects regression analysis was conducted in MATLAB (Mathworks, Natick, MA) to compare the changes in performance as a function of feedback type, removal, and order to compare the changes in performance as a function of multiple factors, i.e.,

(3) $$CCI_{diff}\;\sim \;1+\left(type*removal*order\right)+\left(1|ID\right)+(1|ID:leg)$$

$CCI_{diff}$ denotes the difference between the CCI value for a trial within a feedback testing block and the CCI value from the final baseline trial from that feedback block. The fixed effects include the intercept (‘1’), the type of feedback (type), the removal of feedback (removal), and the order of the type of feedback the participant received (order). While feedback order was not included in the hypotheses, past research has demonstrated that there is an interaction between concurrent and terminal feedback (see, for example, [15]). However, the nature of that interaction is not well understood. Furthermore, trial number was not included in this model because likelihood ratio tests indicated that adding trial number did not significantly improve the model. The first random effects variable (1|ID) accounts for differences between participants (ID). The second random effects variable (1|ID:leg) is an interaction term between participant and which leg the CCI value comes from, accounting for participants potentially favoring one side. Planned contrasts to evaluate the hypotheses were then conducted with F-tests, and the *p*-values for the resulting coefficients and contrasts were evaluated at a significance level of α=0.05.

## 3. Results

### 3.1. Reliability Analysis Results

The ICC values for each combination of normalization technique and CCI definition are documented in Table 2. Except $CCI_{RL}$–mean for the right ankle, all ICC values were excellent. Interestingly, the $CCI_{FW}$ definition was nominally the same compared to when the sEMG signals were normalized by the mean value, whereas the $CCI_{RL}$ definition was more reliable when the sEMG signals were normalized by the peak values. Specifically, note that $CCI_{RL}$–mean had a wider interval, with a lower upper bound, which indicates less precision in the estimate and lower similarity in the estimate.

### 3.2. Linear Mixed-Effects Regression Results

Given the results in the previous subsection, the linear mixed-effects regression results presented in this section are for $CCI_{FW}$ and $CCI_{RL}$ calculated with sEMG signals that were normalized by peak values. For the interested reader, the results for $CCI_{FW}$ and $CCI_{RL}$ calculated with sEMG signals that were normalized by mean values are documented in Appendix A. Table 3 and Table 4 report the results for $CCI_{FW}$ and $CCI_{RL}$, respectively. The adjusted $R^{2}$ values for the $CCI_{FW}$ and $CCI_{RL}$ linear mixed-effects models were 0.29 and 0.33, respectively. Unsurprisingly, the linear mixed-effects regression results were different depending on which definition of CCI was used. Note, the trends in the coefficients were consistent between the two CCI definitions. Interestingly, there were no significant results from the FW definition of CCI that were not also present in the results from the RL definition of CCI. These results are likely due to the two definitions of CCI providing some redundant information, but they are also, to a certain extent, complementary. This conclusion is evidenced by the moderate correlation coefficient between $CCI_{FW}$ and $CCI_{RL}$ (r=0.70), which accordingly implies that only about half of the variance in either CCI can be explained by the other.

**Figure 3:**
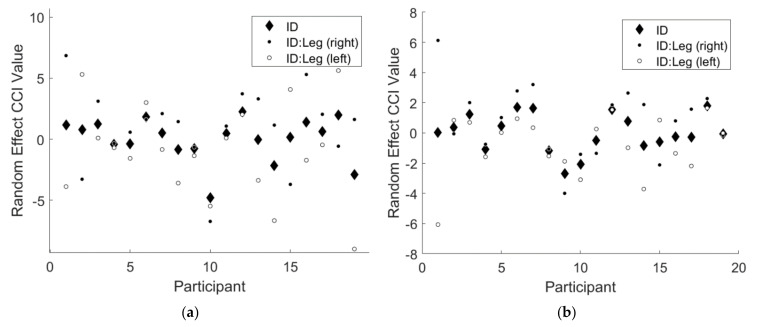
An illustration of the significant random effects from the linear mixed effects regression model for (**a**) $CCI_{FW}$ and (**b**) $CCI_{RL}$. The solid black diamonds denote the random effect of participant, the solid black circles denote the random effect of right leg for each participant, and the solid white circles denote the random effect of left leg for each participant.

Note that the 95% confidence intervals for both random effects terms did not include 0 for either CCI results. This finding implies there was a significant random effect of participant for both CCI results, indicating that some participants responded differently to the two types of feedback. Additionally, there was a significant random effect of the interaction of participant and leg, indicating that some participants favored one leg over the other. Figure 3 below illustrates the magnitudes of these random effects.

To assist in interpretation, the tables below document the predicted values for each combination of the fixed main and interaction effects. Table 5 contains the linear mixed-effects model predictions for $CCI_{FW}$ and Table 6 contains the linear mixed-effects model predictions for $CCI_{RL}$.

**Figure 4:**
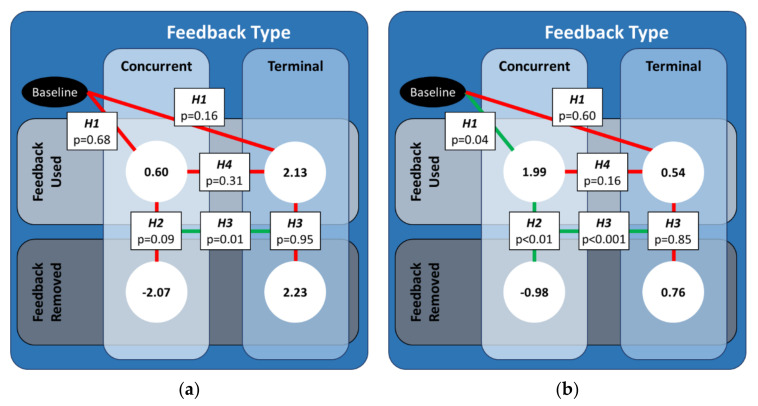
An illustration of the significant differences between the predicted values of CCI-diff for both feedback types for the case when that feedback type was used first. The values in the white circles are the predicted values from the linear mixed effects regression model for (**a**) $CCI_{FW}$ and (**b**) $CCI_{RL}$. Solid green (red) lines denote the cases for which the coefficients from the linear mixed-effect regression were (not) statistically significant.

The predictions revealed a significant interaction between the type of feedback (*type*), the removal of feedback (*removal*), and the order of the type of feedback the participant used (*order*), which is further illustrated and discussed in Appendix B. However, the hypotheses developed for this study focused specifically on the relationship between feedback type and removal. Thus, we concentrated on the effects for the concurrent (terminal) feedback testing block when concurrent (terminal) feedback was used first. Figure 4 below illustrates the statistical results of the planned contrasts and their mappings to the hypotheses.

For the FW definition of CCI, the CCI values did not significantly change when either type of feedback was used, nor was there a significant difference between CCI values when concurrent feedback was used relative to when terminal feedback was used. While there were no significant differences in CCI values when either type of feedback was removed, there was a significant difference in how much the CCI values changed when concurrent feedback was removed compared to when terminal feedback was removed.

For the RL definition of CCI, the CCI values significantly increased when concurrent feedback was used, but there was no significant change when terminal feedback was used. However, the CCI values when concurrent feedback was used were not significantly different from the CCI values when terminal feedback was used. The CCI values significantly decreased when concurrent feedback was removed, whereas the CCI values did not significantly change when terminal feedback was removed. As such, there was also a significant difference in how much the CCI values changed when concurrent feedback was removed compared to when terminal feedback was removed.

## 4. Discussion

Excellent or good reliability was demonstrated by all combinations of CCI definition and normalization technique for both ankles individually and averaged. The confidence interval widths and bounds were observed to be similar for both normalization techniques for the FW CCI definition. However, the RL CCI definition had a narrower confidence interval and, thus, the similarity across the baseline trials was more consistent when the sEMG signals were normalized by the peak muscle activity value. While the linear mixed-effects regression analysis was conducted on all four combinations (Table 3, Table 4, Table A1 and Table A2), only the results utilizing the peak normalization technique were documented above and will be discussed in detail next.

With the CCI interpretations described in Section 2.4 in mind, let us now reconsider the results of the linear mixed-effects regression analyses. As a reminder, the two definitions of CCI utilized in this study are complementary. The FW definition of CCI suggests the relationship between the agonist–antagonist muscle pair changed, whereas the RL definition of CCI suggests that the relationship changed and/or the overall muscle activation of both muscles changed. The results common to both CCI definitions imply that the agonist–antagonist muscle relationship changed, which will be referred to as ankle co-contraction recruitment. The results limited to the RL definition implies the overall muscle activation increased for both muscles relatively uniformly, which will be referred to as ankle co-contraction modulation. 

The first hypothesis (**H1**) asserted that ankle co-contraction would increase relative to baseline when either type of feedback was used. In support of this hypothesis, the planned contrast revealed ankle co-contraction modulation (RL) significantly increased only when concurrent feedback was used. The second hypothesis (**H2**) asserted that ankle co-contraction would decrease when concurrent feedback was removed relative to when the feedback was used. In support of this hypothesis, the contrast revealed the ankle co-contraction modulation (RL) significantly decreased when concurrent feedback was removed. The third hypothesis (**H3**) asserted that decreases in ankle co-contraction when terminal feedback was removed would be less than the decreases in ankle co-contraction when concurrent feedback was removed. In opposition to this hypothesis, one of the contrasts revealed neither ankle co-contraction recruitment (FW&RL) nor modulation (RL) changed when terminal feedback was used or when it was removed. However, in support of this hypothesis, another contrast revealed that the changes in ankle co-contraction recruitment (FW&RL) and modulation (RL) were different when concurrent feedback was removed as compared to when terminal feedback removed. The final hypothesis (**H4**) asserted that increases in ankle co-contraction relative to baseline would be smaller for terminal feedback than for concurrent feedback. In opposition to this hypothesis, the contrast revealed that neither the ankle co-contraction recruitment (FW&RL) nor modulation (RL) was significantly different between the two feedback types when feedback was used. 

Here, we provide a brief discussion of a subset of the aforementioned results to elucidate the importance of utilizing both definitions of CCI. The relative ratio of the agonist–antagonist muscle activation remained nominally the same when concurrent feedback was used. Simultaneously, the absolute magnitude of the agonist–antagonist muscle activation increased. Since the relative activation ratio did not change while the absolute activation magnitude did, this result implies an overall increase in ankle stiffness. It should be noted that this conclusion could only be reached with the results from both CCI definitions. Focusing on the relative ratio for ankle co-contraction recruitment (FW) cannot provide information about joint stiffness, as has been demonstrated by previous investigations [39,40]. Similarly, focusing on the absolute magnitude for ankle co-contraction modulation (RL) cannot provide information about whether changes were driven by the relative ratio or absolute magnitude in muscle activation.

In the previous preliminary study [32] that evaluated the effects of feedback on outcome measures of standing balance performance involving the same kinematic data used in this study to generate the feedback, trunk sway angles as measured by root mean square of the angular displacements (RMS) and areas of 95th percentile confidence interval elliptical fit to the sway data (EA) significantly decreased when either type of feedback was used. However, the decreases observed when participants used terminal feedback were significantly less than that those observed when participants used concurrent feedback. Mean sway velocities (MV) also significantly increased when participants used concurrent feedback, but significantly decreased when participants used terminal feedback. Combined with the results of this study, these collective findings imply that when participants used concurrent visual feedback, a significant increase in ankle co-contraction modulation coincided with significant decreases in sway angles and significant increases in sway velocity. When participants used terminal feedback, no significant changes in ankle co-contraction recruitment or modulation were observed, which coincided with significant decreases in sway angles and sway velocity. When concurrent feedback was removed, the sway angles (velocities) increased (decreased) to return to their baseline levels. The significant increase in sway angles and significant decrease in sway velocities coincided with a significant decrease in ankle co-contraction modulation. Removing terminal feedback did not have a significant effect on the sway angles or sway velocities, which coincided with no significant changes in ankle co-contraction recruitment or modulation.

Although sEMG data from trunk muscle groups (e.g., external obliques and paraspinals) were not measured in this study, past literature suggests that hip or mixed (e.g., ankle and hip [41] or ankle, hip/trunk, and arms [8]) strategies are alternative or supplemental approaches to an ankle strategy. The ankle muscle sEMG results from this study combined with the trunk-mounted IMU results from the previous preliminary investigation [32] imply a different strategy was likely learned or applied by participants using terminal feedback as compared to those using concurrent feedback. This difference in strategy is further evidenced by the significant interaction between the type of feedback, the removal of feedback, and the order of the type of feedback the participant used explored more extensively in Appendix B. For example, Kim and Hwang (2018) showed that young healthy participants adopted a hip or mixed strategy in addition to increasing ankle co-contraction recruitment when their standing balance was perturbed [41]. When participants with bilateral peripheral vestibular deficits used multimodal concurrent feedback for sway angles, the significant reduction in sway angles coincided with reduced EMG activity levels in bilateral muscles in the trunk as well as the ankle [8,12]. Young healthy participants who used concurrent visual feedback for their medial gastrocnemius muscle activation level to achieve a specific range of muscle activity exhibited significant increases in center of pressure deviations that coincided with increasing muscle activation level [25]. At higher muscle activity levels (similar to what is expected from standing on a compliant surface [22]), the participants’ movements were more reliant on hip and mixed strategies [25]. When concurrent visual feedback of center of pressure was used by young healthy adults, center of pressure deviations decreased, whereas the frequencies increased [10,43]. dos Anjos et al. [10] attributed the accompanying decrease in ankle angle deviations and increase in ankle angle deviation frequency to an overall increase in ankle stiffness, though they noted that a hip strategy could have been present as well. In another study, young healthy participants voluntarily reduced their center of mass sway, which was associated with increases in ankle co-contraction [24]. However, changes in ankle co-contraction did not correlate with sway reduction for individual participants, which implies this strategy was not always successful. Older adults have been known to increase ankle co-contraction (using the RL definition) as a nondiscriminatory, general strategy to reduce overall sway [5,25]. This implication is particularly noteworthy when considering the increased fall risk that is associated with elevated ankle co-contraction modulation [29].

The results of this study, as well as those from the previous study [32], imply that concurrent visual feedback of body angles may prompt participants to adopt a dominant ankle strategy that is generally considered to be maladaptive for older adults. On the other hand, terminal visual feedback may prompt participants to adopt a hip or mixed strategy to achieve the aforementioned improvements in sway performance that did not coincide with significant changes in ankle co-contraction recruitment or modulation. Future work should consider hip and trunk muscle activity to characterize whole body kinematic responses to concurrent and visual feedback. Balance training that utilizes concurrent visual feedback may benefit from additional, multimodal forms of feedback that also convey ankle muscle activity information to increase the use of a hip or mixed strategy. For example, auditory concurrent feedback of EMG activity levels has been described as being potentially useful for re-education of muscle activation during certain tasks [7], though EMG-based feedback may be inappropriate for certain pathological populations, like those with Parkinson’s disease [25]. Alternatively, terminal visual feedback has less significant real-time and short-term gains in sway performance, but better long-term post-training performance, typically achieved by hip or mixed strategies (i.e., not a dominant ankle strategy). The choice of concurrent or terminal feedback for balance training applications should consider the total exposure time to the intervention; for example, concurrent feedback might be most appropriate for single-session, short-term uses, whereas terminal feedback might be most appropriate for training programs spanning a longer period of time. Beyond balance control strategies, potential changes in sensorimotor-related factors (e.g., sensory reweighting) and patient-usability-related factors, among other factors, should be considered when selecting a form of feedback to pair with a balance training intervention [44,45].

This study has several limitations, including a relatively small sample size, conducting a single standing balance task, and a single training session for both feedback modalities. The findings of this study suggest that, for single use of sporadic balance training, terminal visual feedback encourages a less dominant ankle strategy that reduces sway compared to concurrent visual feedback. However, prior studies have noted changes in feedback use over time, finding that feedback may interfere with a task, even until the third day of training [46,47]. The effects of concurrent feedback may, therefore, change over extended training. Similarly, long-term retained changes in balance strategy or balance strategies in related but different balance tasks may or may not follow the same trends as seen here. Future work should, therefore, consider the effects of feedback type when utilized over multiple training sessions and explore carry over and retention of the training. Finally, many previous investigations found hip or mixed strategies in addition to or in place of the ankle strategies studied in this investigation. Thus, future work should also assess which strategy might be dominant.

## 5. Conclusions

This preliminary study investigated the effects of concurrent and terminal visual feedback during a standing balance task on ankle co-contraction, which was captured by two complementary mathematical definitions. Following a novel analysis of CCI reliability as a function of the sEMG normalization technique, linear mixed-effects regression analyses and planned contrasts revealed participants developed different strategies depending on if they received concurrent or terminal feedback. Ankle co-contraction modulation increased when concurrent feedback was used and significantly decreased when concurrent feedback was removed. Ankle co-contraction recruitment and modulation did not significantly change when terminal feedback was used or when it was removed. Neither ankle co-contraction recruitment nor modulation were significantly different between the two feedback modalities when they were used. However, the changes in ankle co-contraction recruitment and modulation were different when concurrent feedback was removed as compared to when terminal feedback removed. Finally, this study found a significant interaction between between the type of feedback, the removal of feedback, and the order of the type of feedback the participant used. The findings from this study have implications for the design of balance training technologies using visual feedback, and indicate the need for additional research to understand how different feedback modalities encourage different balance strategies.

## Figures and Tables

**Table 1:** Participant demographics by feedback testing block assignment. Note, all participants used both types of feedback.

	Concurrent Feedback Testing Block First	Terminal Feedback Testing Block First
Male/Female	3/7	4/5
Age (STD) [years]	70.4 (3.0)	70.0 (3.0)

**Table 2:** Interclass coefficients (ICCs) and the corresponding 95% confidence intervals (95% CI) for both ankles for all four combinations of co-contraction index (CCI) definition (FW or RL) and normalization technique (peak or mean). The averages of both ankles’ ICC values for each CCI definition and normalization technique combination are also included.

	Left Ankle		Right Ankle		Average
	ICC	95% CI	ICC	95% CI	ICC
$CCI_{FW}$–peak	0.96	[0.92, 0.98]	0.95	[0.90, 0.98]	0.96
$CCI_{FW}$–mean	0.98	[0.96, 0.99]	0.98	[0.95, 0.99]	0.98
$CCI_{RL}$–peak	0.95	[0.90, 0.98]	0.98	[0.96, 0.98]	0.97
$CCI_{RL}$–mean	0.92	[0.84, 0.96]	0.88	[0.77, 0.94]	0.90

**Table 3:** Summary of linear mixed-effects regression results for $CCI_{FW}$. CI:LB and CI:UB denote the lower and upper bounds of the 95% confidence intervals for the estimated coefficient. The options in parentheses are the baseline (comparison) categories.

	Coefficient	CI:LB	CI:UB	*p*-Value
**Main Fixed Effects**				
(Intercept)	0.60	−2.20	3.40	0.68
*type* (concurrent)	−1.25	−2.98	0.47	0.15
*removal* (feedback used)	−2.67	−4.79	−0.55	0.01 *
*order* (concurrent first)	4.78	0.70	8.85	0.02 *
**Fixed Interaction Effects**				
*type:removal*	0.74	−2.28	3.77	0.63
*type:order*	−2.00	−4.51	0.51	0.11
*removal:order*	−6.35	−9.43	−3.28	**<0.001 ^‡^**
*type:removal:order*	8.38	3.98	12.78	**<0.001 ^‡^**
**Random Effects**				
*ID*	2.69	1.09	6.67	-
*ID:leg*	4.30	2.99	6.18	-

Significant at α = * 0.05, ^‡^ <0.001.

**Table 4:** Summary of linear mixed-effects regression results for $CCI_{RL}$. CI:LB and CI:UB denote the lower and upper bounds of the 95% confidence intervals for the estimated coefficient. The options in parentheses are the baseline (comparison) categories.

	Coefficient	CI:LB	CI:UB	*p*-Value
**Main Fixed Effects**				
(Intercept)	1.99	0.08	3.89	0.04 *
*type* (concurrent)	−3.30	−4.56	−2.04	**<0.001 ^‡^**
*removal* (feedback used)	−2.97	−4.52	−1.42	**<0.001 ^‡^**
*order* (concurrent first)	5.81	3.04	8.57	**<0.001 ^‡^**
**Fixed Interaction Effects**				
*type:removal*	2.07	−0.14	4.28	0.07
*type:order*	−3.96	−5.79	−2.13	**<0.001 ^‡^**
*removal:order*	−6.14	−8.39	−3.90	**<0.001 ^‡^**
*type:removal:order*	7.26	4.05	10.47	**<0.001 ^‡^**
**Random Effects**				
*ID*	1.88	0.81	4.33	-
*ID:leg*	2.77	1.90	4.04	-

Significant at α = * 0.05, ^‡^ <0.001.

**Table 5:** Predicted differences in ankle co-contraction ($CCI_{FW}$) relative to baseline using the results from the linear mixed-effects regression model described in Table 2.

	Concurrent Feedback Testing Block First		Terminal Feedback Testing Block First	
	Concurrent	Terminal	Concurrent	Terminal
**Feedback Used**	0.60	−0.65	5.38	2.13
**Feedback Removed**	−2.07	−2.58	−3.64	2.23

**Table 6:** Predicted differences in ankle co-contraction ($CCI_{RL}$) relative to baseline using the results from the linear mixed-effects regression model described in Table 3.

	Concurrent Feedback Testing Block First		Terminal Feedback Testing Block First	
	Concurrent	Terminal	Concurrent	Terminal
**Feedback Used**	1.99	−1.31	7.8	0.54
**Feedback Removed**	−0.98	−2.21	−1.31	0.76

**Figure A1:**
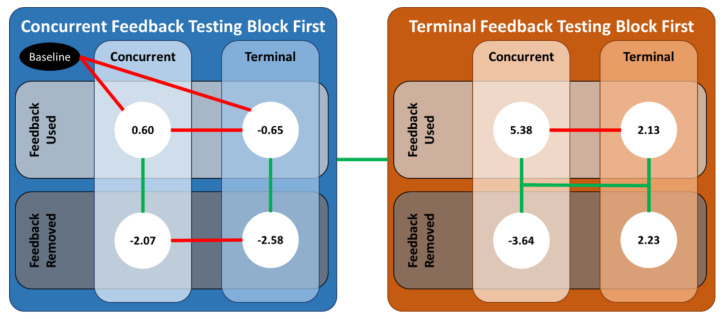
An illustration of the significant differences between the various combinations of the $CCI_{FW}$ predictor variables. The values in the white circles are the predicted values from the linear mixed-effects regression model for $CCI_{FW}$. Solid green (red) lines denote the case for which the coefficients from the linear mixed-effect regression were (not) statistically significant.

**Figure A2:**
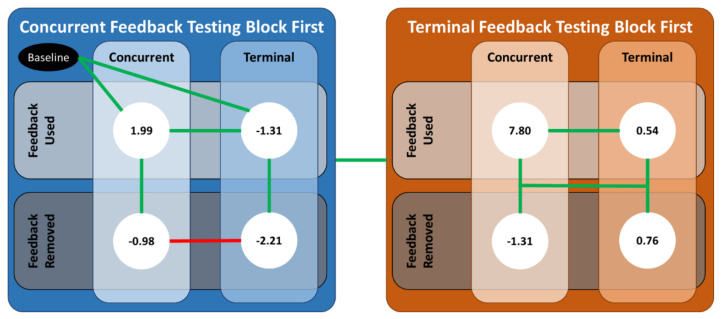
An illustration of the significant differences between the various combinations of the $CCI_{RL}$ predictor variables. The values in the white circles are the predicted values from the linear mixed-effects regression model for $CCI_{RL}$. Solid green (red) lines denote the case for which the coefficients from the linear mixed-effect regression were (not) statistically significant.

## Data Availability

The de-identified datasets may be available upon reasonable request.

## Appendix A

Table A1 and Table A2 contain the linear mixed-effect regression results for the co-contraction indices (CCI) calculated via the definition offered by Falconer and Winter ($CCI_{FW}$) and by Rudolph/Lewek ($CCI_{RL})$, respectively. The CCI values were calculated with sEMG signals normalized by mean values. The linear mixed-effects regression results for both CCI definitions calculated with sEMG values normalized by mean muscle activation differed from each other as well as the peak normalization counterparts. For $CCI_{FW}$, only the removal of feedback fixed-effect term and the interaction between feedback removal, type, and order fixed-effect term were statistically significant. For $CCI_{RL}$, only the interaction term between feedback removal and type was not statistically significant. Interestingly, this term was insignificant across all four CCI definition and normalization technique pairs. Additionally, the magnitudes of the coefficients in Table A2 were much larger than the magnitudes of the coefficients for the other three pairs (by about a factor of 10 in most cases).

**Table A1:** Summary of linear mixed-effects regression results for $CCI_{FW}$. CI:LB and CI:UB denote the lower and upper bounds of the confidence intervals for the estimated coefficient. The options in parentheses are the baseline (comparison) categories.

	Coefficient	CI:LB	CI:UB	*p*-Value
**Main Fixed Effects**				
(Intercept)	−0.34	−2.81	2.14	0.79
*type* (concurrent)	−1.02	−2.46	0.42	0.16
*removal* (feedback given)	−2.17	−3.93	−0.41	**0.01 ***
*order* (concurrent first)	1.21	−2.40	4.81	0.51
**Fixed Interaction Effects**				
*type*removal*	−0.74	−3.25	1.78	0.57
*type*order*	1.75	−0.34	3.84	0.10
*removal*order*	−2.24	−4.80	0.32	0.09
*type*removal*order*	5.67	2.00	9.33	**<0.01 ^†^**
**Random Effects**				
ID	2.78	1.47	5.27	-
ID:leg	3.32	2.29	4.81	-

Significant at α = *** 0.05, ^†^ <0.01**.

**Table A2:** Summary of linear mixed-effects regression results for $CCI_{RL}$. CI:LB and CI:UB denote the lower and upper bounds of the confidence intervals for the estimated coefficient. The options in parentheses are the baseline (comparison) categories.

	Coefficient	CI:LB	CI:UB	*p*-Value
**Main Fixed Effects**				
(Intercept)	12.81	2.48	23.13	**0.02 ***
*type* (concurrent)	−18.92	−25.28	−12.56	**<0.001 ^‡^**
*removal* (feedback given)	−20.09	−27.87	−12.30	**<0.001 ^‡^**
*order* (concurrent first)	22.54	7.54	37.54	**<0.01 ^†^**
**Fixed Interaction Effects**				
*type*removal*	10.09	−1.04	21.22	0.08
*type*order*	−14.49	−23.72	−5.25	**<0.01 ^†^**
*removal*order*	−24.13	−35.44	−12.81	**<0.001 ^‡^**
*type*removal*order*	36.12	19.94	52.31	**<0.001 ^‡^**
**Random Effects**				
ID	11.08	5.51	22.30	-
ID:leg	14.26	9.81	20.72	-

Significant at α = *** 0.05, ^†^ <0.01, ^‡^ <0.001**.

Table A3 and Table A4 below document the predicted values for each combination for the fixed main and interaction effects. Specifically, Table A3 contains the results for the linear mixed-effects model from Table A1 and Table A4 contains the results for the linear mixed-effects model from Table A2.

**Table A3:** Predicted differences in ankle co-contraction relative to baseline using the results from the linear mixed-effects regression model described in Table A1.

	Concurrent First		Terminal First	
	Concurrent	Terminal	Concurrent	Terminal
**Feedback Used**	1.99	−1.31	7.8	0.54
**Feedback Removed**	−0.98	−2.21	−1.31	0.76

**Table A4:** Predicted differences in ankle co-contraction relative to baseline using the results from the linear mixed-effects regression model described in Table A2.

	Concurrent First		Terminal First	
	Concurrent	Terminal	Concurrent	Terminal
**Feedback Used**	12.81	−6.11	35.35	1.94
**Feedback Removed**	−7.28	−16.11	−8.87	3.93

## Appendix B

There were a few notable differences that have potentially important implications for interpretation of the effects of concurrent and terminal feedback. Figure A1 and Figure A2 below illustrate which of these conditions were significant given how the linear mixed-effects regression models (Table 3 and Table 4 for $CCI_{FW}$ and $CCI_{RL}$, respectively) were developed. Note that Figure A1 and Figure A2 illustrate the results from the linear mixed-effects regression models, whereas Figure 4 illustrated the results from the planned contrasts.

Starting with the common results, ankle co-contraction recruitment decreased when either type of feedback was removed for participants who received concurrent feedback first, which is evidence in support of **H2**. Specifically, when the feedback was removed, the participant relied significantly less on the antagonist muscle to stiffen the ankle joint. To our knowledge, this is the first time that it has been demonstrated that older adults can reduce their ankle co-contraction recruitment relative to baseline with visual (more so for terminal than concurrent) feedback displaying center of mass sway angles. For example, the participants in [7] were significantly younger than this study’s population, and they achieved reduced levels of muscle activity while receiving sEMG auditory feedback.

Next, there were conflicting results depending on whether participants received concurrent or terminal feedback first. For the former group, the decrease in ankle co-contraction recruitment when the feedback was removed was not significantly different between the two modalities, which opposes **H3**. However, for the latter group, who received terminal feedback first, ankle co-contraction recruitment did not meaningfully change when terminal feedback was removed, whereas it decreased significantly when concurrent feedback was removed, which supports **H3**. These conflicting results are offered in support of a potentially meaningful interaction between concurrent and terminal visual feedback. The predicted estimates for both CCI definitions imply the ankle co-contraction recruitment did, on average, increase when terminal feedback was used first. Older adults have been known to increase ankle co-contraction as a nondiscriminatory, general strategy to reduce overall sway [5,24,25]. It is possible that by receiving terminal feedback first, participants learned and/or adopted a strategy to increase ankle stiffness through antagonist muscle activation. Then, this strategy was heightened when used with concurrent feedback, which is evidenced by the large increase in ankle co-contraction recruitment when concurrent feedback was given thereby supporting **H4**.

For the results specific to the RL CCI definition, ankle co-contraction modulation increased when concurrent feedback was received more than when terminal feedback was received, regardless of feedback order, which further supports **H4**. Along with the ankle co-contraction recruitment results, these collective results imply that participants increased both agonist and antagonist muscle activity when concurrent feedback was received, and the agonist muscle contributed more when concurrent feedback was received after terminal feedback. Next, ankle co-contraction modulation increased when concurrent feedback was received first, which supports **H1**. However, ankle co-contraction modulation decreased relative to baseline when terminal feedback was used after concurrent feedback, which opposes **H1**. Together, these results seem to imply that the participants may have used another strategy to reduce their body sway that was not limited to the ankle. For example, the participants in [12] achieved superior balance performance with vibrotactile and auditory concurrent feedback that coincided with reduced muscle activation in the back muscles, as well as those in the ankle.
